# Effect of the Xuebijing oral solution on survival of rabbits with acute paraquat poisoning

**DOI:** 10.1186/1471-227X-12-S1-A6

**Published:** 2012-12-18

**Authors:** Song Zu-jun, Chen Gao-fei, Ao Guo-kun, Cao Jian-xiao

**Affiliations:** 1 Department of Emergency The 309th Hospital of Chinese People’s Liberation Army,17 Heishanhu Road, Haidian District,Beijing 100091, People’s Republic of China; 2 The Fourth Military Medical University; No.127 Changle Xilu Road Xi'an 710032, People’s Republic of China; 3 Department of Rodiology The 309th Hospital of Chinese People’s Liberation Army, 17 Heishanhu Road, Haidian District, Beijing 100091, People’s Republic of China

## Objective

To study the effect of the Xuebijing oral solution on survival of rabbits with acute paraquat poisoning.

## Methods

30 New Zealand white rabbits were randomly divided into saline control group(group A) and two treatment groups of 3ml/kg(group B) and 10ml/kg(group C) of Xuebijing oral solution.All the rabbits were treated with PQ at 35mg/kg through intraperitoneal injection, which would build the model of acute poisoning. Group B and C were treated by gavage with 3ml/kg and 10ml/kg of Xuebijing oral solution respectively, once a day to maintain. Group A were treated with saline instead.

## Results

The analysis of survival curve showed that the survival rate was not significantly different between group A and B, but group C was significantly higher than group A and B(P<0.05).

## Conclusion

Large doses of Xuebijing oral solution may improve the survival rate of acute paraquat poisoning.

